# Four-Stage Audit Demonstrating Increased Uptake of HIV Testing in Acute Neurology Admissions Using Staged Practical Interventions

**DOI:** 10.1371/journal.pone.0134574

**Published:** 2015-09-03

**Authors:** Dilraj Singh Sokhi, Chantal Oxenham, Rebecca Coates, Mhairi Forbes, Nadi K. Gupta, Daniel J. Blackburn

**Affiliations:** 1 Department of Clinical Neurology, Royal Hallamshire Hospital, Sheffield Teaching Hospitals NHS Foundation Trust, Sheffield, South Yorkshire, United Kingdom; 2 Department of Genito-Urinary Medicine, Royal Hallamshire Hospital, Sheffield Teaching Hospitals NHS Foundation Trust, Sheffield, United Kingdom; 3 Sheffield Institute for Translational Neuroscience, University of Sheffield, Sheffield, South Yorkshire, United Kingdom; Imperial College London, UNITED KINGDOM

## Abstract

**Background:**

UK National Guidelines (UKNG) advise HIV testing in clinically indicated neurological presentations. We audited the impact of our practical strategies to increase uptake of HIV testing at a regional acute neurology admissions unit.

**Methods:**

We audited HIV testing in 4 periods over 2 years: before we designed a UKNG-based “HIV testing in Neurology” protocol (“pre-protocol”); after dissemination of the protocol alone (“post-protocol”); post-protocol dissemination combined with both a tailored departmental admissions clerking proforma to prompt for HIV testing & consenting, and regular focussed tutorials to doctors on HIV testing in neurological patients (“post-proforma”); and finally one year after the post-proforma period (“+1 year”). We also looked at the total number of HIV tests sent from the unit during the two-year period. We assessed significance using Fisher’s exact test.

**Results:**

47.8% of all acute neurology non-stroke admissions were eligible for HIV testing during all the audit periods. Testing rates were as follows: pre-protocol 21.9%; post-protocol 36.6%; post-proforma 83.3%; and at +1 year 65.4% (p<0.05 for both post-protocol and +1 year when compared to pre-protocol). Documentation of consent for HIV testing improved from 25% to 67.6% with the HIV-tailored clerking proforma. The total number of HIV tests requested from the unit doubled in the post-proforma period compared to pre-protocol (p<0.05).

**Conclusion:**

In conclusion: the combination of an HIV testing protocol, a tailored departmental clerking proforma and regular focussed teaching to doctors on indications for HIV testing led to a sustained increase in HIV testing uptake in our regional acute neurology admissions unit.

## Introduction

The prevalence of Human Immunodeficiency Virus (HIV) infection in the UK has increased by 25% over five years to an estimated 98,400 people [1]. Early diagnosis is vital as commencing combined anti-retroviral therapy before severe immunosuppression can lead to a near-normal life expectancy [2, 3]. Early knowledge of HIV status also helps change behaviour that reduces HIV transmission to partners [4, 5], which has sizeable health-cost savings: prevention of one HIV infection saves approximately £0.3 million of public funds [6]. Yet one in five, and in some high-risk groups up to a third, of people with HIV are unaware of their diagnosis in the UK [1]. Those diagnosed “late” with HIV (CD4<350 cells/uL) have a worse prognosis [7], and up to a quarter of deaths can be attributed to missed opportunities by healthcare workers to test at an earlier stage [8, 9].

Opt-out HIV testing in all patients is recommended in the USA [10]. The UK National Guidelines for HIV testing (UKNG) 2008 were developed to help normalise and expand HIV testing across healthcare services [11]. Testing all hospitalised patients for HIV, regardless of local prevalence and risk factors, is feasible and cost-effective in the UK and well accepted amongst patients [12–14]. However, the UKNG advocate routine testing only where local diagnosed prevalence is 2/1000 or greater.

Opt-out routine testing rates in “traditional” settings such as sexual health and ante-natal clinics have increased substantially since the UKNG [15]. There has also been some success in other “non-traditional” settings in the UK and Europe [16–28] (see Table 1).

**Table 1:** Summary of studies aimed at improving HIV testing uptake in “non-traditional” settings.

Study: Setting	Pre-study interventions	Study Periods	Results	HIV +ve
Ellis *et al*, 2011 [16]: acute medical admissions (Newcastle)	Specialist-led seminar and tutorials, pre-test patient leaflet, 1-page HIV proforma	14th September–26th October 2009: Junior doctors did 91.1% of tests; 4th January–19th March 2010: Clinical assistant did 78.9% of tests	478/3753 approached (12.7%); 396/478 consented (82.8%)	2
Palfreeman *et al*, 2013 [17]: Acute Medical Admissions Unit (Leicester)	Consultants emailed, junior doctors’ induction, AMU staff trained	“Pre-pilot”: September 2008–August 2009	205/5484 tested (3.7%)	4
	Additionally: patient posters, multi-lingual leaflets, clerking proforma tailored for HIV testing	“Pilot”: September 2009—August 2010 (weekly visit by HIV physician to AMU)	937/5517 tested (17.0%)	10
		“Post-pilot”: September 2010-August 2011 (no further interventions)	1399/6225 tested (22.5%)	15
Phillips *et al*, 2014 [18]: Acute Medical Admissions Unit (Croydon)	Specialist-led seminar and tutorials, consultants emailed, juniors’ induction, AMU staff trained, intranet updates, patient posters and admission packs, HIV-tailored proforma	Jul 2011 –Mar 2013 Weekly visits to AMU by HIV team for first 3 months	4122/12682 tested (32.5%)	14
Peck *et al*, 2010 [19]: Acute Admissions Unit (UCL, London)	Intranet guideline, consultants informed, trainees tutorial	15th April–18th July 2008	16/56 tested (28.6%)	1
Rayment *et al*, 2012 [20] HIV in non-traditional settings (the HINTS study): all study sites in London	*(Chelsea & Westminster Emergency Department)* Seconded staff, sexual health department support, non-clinical testers	August–November 2009	3433/5505 approached (62.4%); 2121/3433 consented (61.8%)	4
	*(Homerton Acute Care Unit)* Seconded staff from various departments	January–April 2010	548/1298 approached (42.2%); 384/548 consented (70.1%)	4
	*(Kings College Dermatology clinic)* Two student HIV testers offering test pre-clinic	July–September 2010	1329/5352 approached (24.8%); 1002/1329 consented (75.4%)	0
	*(North End Primary Care Medical Centre)* Practice clinical staff had focussed training; testing introduced in a staggered fashion	February—May 2010	884/1700 approached (52%); 598/884 consented (67.6%)	0
Perry *et al*, 2010 [27]: Acute General Medicine (Brighton)		August–December 2009	1190/2735 tested (43.5%)	2
Alston *et al*, 2013 [28]: Acute admissions (Surrey & Sussex)		2011	1% in high-prevalence areas; 3% in low-prevalence areas (out of 200 case notes reviewed)	?

Dissemination of UKNG using conventional methods alone–presenting at medical “grand rounds”, via letter and email, posting guidelines on wards and doctors’ offices–has been shown to have little impact on uptake of HIV testing even in high sero-prevalent areas such as London and Blackpool [19, 22]. More robust publicity, providing multi-lingual patient information leaflets, focussed tutorials for frontline testing staff, admission proforma prompts for HIV testing, regular on-site support by HIV staff, and using trained individuals to obtain consent and blood samples have been shown to be more successful [16–18, 20, 21].

The UKNG clinical indicator conditions include neurological presentations of HIV infection. HIV enters the nervous system early during acute infection and can affect any part of the nervous system; 10% of HIV sero-conversion presentations are neurological, usually as an aseptic meningo-encephalitis [29]. Therefore, a pure neurological presentation of HIV is an opportunity to diagnose the infection at an early stage; indeed, it is recommended to test for HIV in all patients with suspected encephalitis [30].

**Fig 1:**
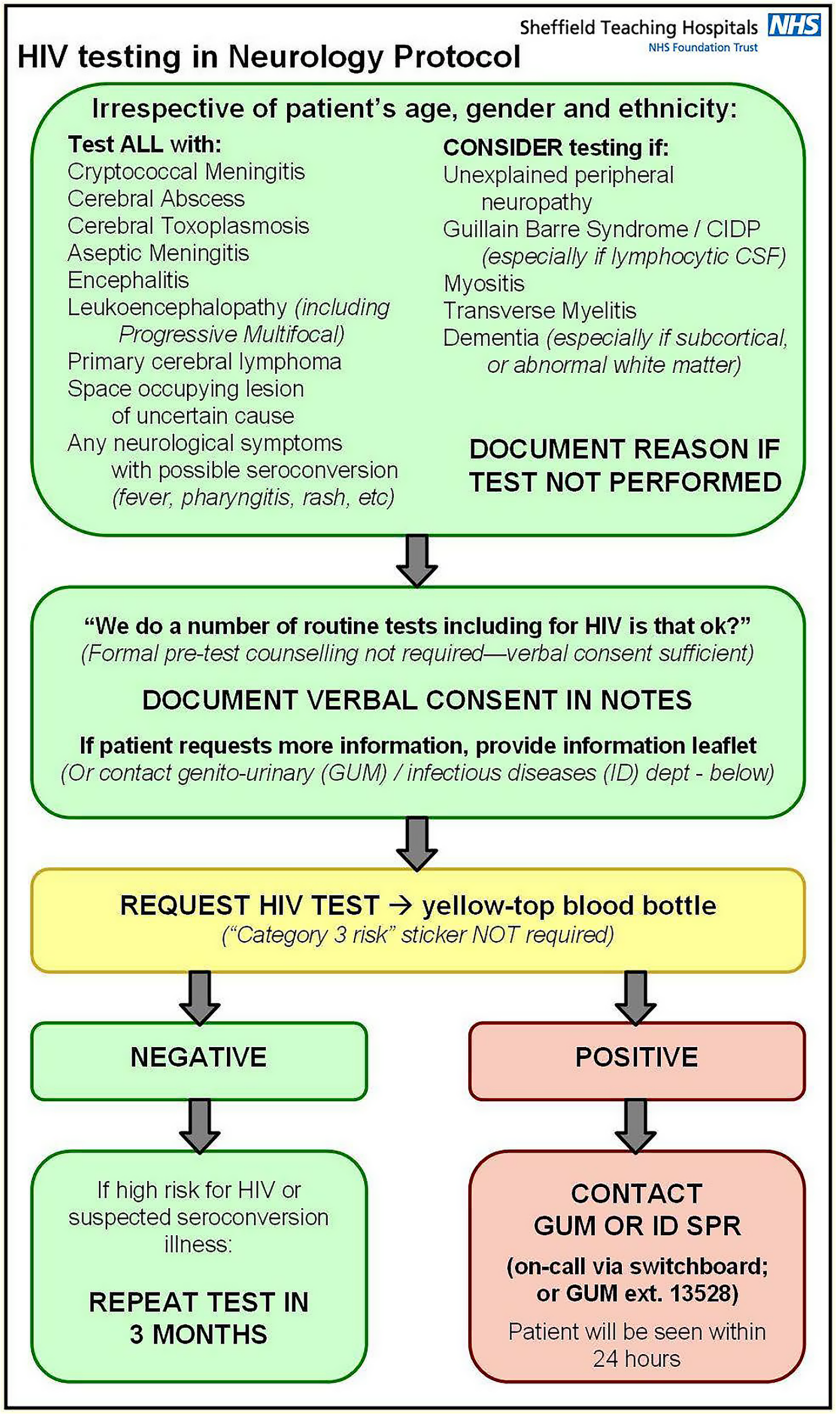
The Sheffield “HIV Testing in Neurology” protocol. Based on the UKNG Guidelines for HIV testing. We have previously presented preliminary data from an audit of the effectiveness of this protocol alone in improving HIV testing [32]. We evaluated efficacy of improving HIV testing in eligible patients by re-auditing our service after changing the neurology admission proforma and introduced training sessions. We also studied the total number of HIV tests sent from the acute neurology unit.

The Department of Clinical Neurology in Sheffield, South Yorkshire, is a tertiary centre which admits patients with acute neurological presentations, including strokes. There are approximately 3,500 acute admissions per year, of which 50–60% are discharged within 48 hours (cases such as uncomplicated exacerbations of seizures, acute headaches, multiple sclerosis relapses and medical decompensation of chronic neurological disorders). The prevalence of HIV in Yorkshire has trebled over the last decade, and Sheffield’s HIV prevalence has risen from 1.4 to 1.8/1000 [31]. In late 2010 a middle-aged patient was transferred from a district general hospital, but the HIV-related diagnosis causing the neurological decline was not realised until three months later, at which point their CD4 count was 13. This prompted collaboration with the local Genito-Urinary Medicine department to improve HIV testing in eligible acute neurology patients, and a local “HIV Testing in Neurology” protocol was devised, based on the UKNG (Fig 1).

## Materials and Methods

We prospectively reviewed medical notes for patients admitted as acute non-stroke neurology cases over four time periods:

1. “Pre-protocol”, 30^th^ July–31^st^ August 2012: before the protocol was first disseminated to clinical staff;
2. “Post-protocol”, 19^th^ October–10^th^ November 2012: after the protocol was emailed to all departmental consultants and frontline staff, and copies placed on ward noticeboards, in doctors’ offices and on notes trolleys;
3. “Post-proforma”, 10^th^ June 2013–1^st^ August 2013: 2 months after altering the neurology departmental clerking proforma to prompt on consenting and testing for HIV in eligible patients. We did this by adding the questions “Is HIV testing indicated? Yes/No” and “If indicated, consent obtained? Yes/No” into the mandatory statements section (this section also includes prompts for venous thromboprophylaxis, decision for resuscitation procedures and cognitive screening). Implementation of the new proforma in April 2013 coincided with the rotation of new junior doctors starting at the hospital. At the hospital induction, we educated doctors about the importance of HIV testing in acute neurology patients and reinforced this with a focussed tutorial in May 2013.
4. “+ 1 year”, 28^th^ May–20^th^ July 2014: we continued with the interventions of stage (3.) above every doctors’ rotation, and re-audited at the end of one year.

We aimed to review a minimum of 60 case-notes during each audit period after the protocol was disseminated, and collected data as to whether:

1. the patient was eligible for an HIV test;
2. the HIV test was undertaken, and if so what the result was;
3. consent was documented;
4. the eventual diagnosis was an indicator condition.

In addition, in order to measure the effect of our interventions on overall uptake of HIV testing within our acute neurology service, we cross-referenced all HIV tests undertaken within the hospital Trust, held on a database by the Department of Virology, with our electronic database of all acute admissions including stroke for the period 1^st^ June 2012–31^st^ August 2014.

All data were inputed and analysed on Microsoft Excel.

### Ethical Approval

The project was a service evaluation and quality improvement initiative by the Sheffield Teaching Hospitals NHS Foundation Trust Clinical Effectiveness and Audit Unit (CEAU). Formal ethical approval by the local research ethics board was therefore not required.

### Patient Consent

We strictly adhered to the UKNG for patient consent during HIV testing: indications for HIV testing were explained to all patients before undertaking the test, and for those patients who lacked capacity at the time of testing, a medical decision was made based on the patient’s best interests. As per UKNG guidelines, consent was documented in the acute admissions clerking proforma using “Yes/No” answers. For those patients who lacked capacity to consent for HIV testing but in whom testing was definitely indicated, the test was still undertaken by the consultant’s team if it was deemed to be in the best interests of the patient.

The CEAU indicated that specific consent for this service evaluation study was not required as we were following local Trust guidelines on information governance and patient confidentiality including in the context of “sensitive diseases” such as HIV. The procedures for consent for HIV testing as above were accepted by the CEAU.

We followed the SQUIRE Guidelines (www.equator-network.org) throughout this quality improvement study. The project was in keeping with the principles of the Declaration of Helsinki.

## Results

There were 6723 admissions over 2 years and 2 months: 4349 (64.6%) were acute non-stroke neurology admissions, 928 (13.8%) were acute stroke admissions, and the rest were elective admissions. All patients approached for HIV testing accepted to have the test, giving a patient uptake rate of 100%. There were no positive HIV tests throughout the audit period.

Table 2 summarises the results of the audit, and the diagnoses of patients in whom HIV test was indicated are shown in Table 3. “Post-protocol” testing of eligible patients was higher at 36.6% compared to 21.9% during the “pre-protocol” period, and highest during the “post-proforma” period at 83.3% but this reduced to 65.4% at “+1 year”. Overall HIV testing rates during the casenotes audit periods (and for all admissions) consistently increased through the two years, from 12.3% (9.0% for all admissions) during “pre-protocol”, to 18% (20.4%) during “post-protocol”, then to 33.9% (23.4%) during “post-proforma” and finally to 28.3% at “= 1 year”. 70 of the 80 eligible patients during the casenotes audit period had UKNG clinical indicator diseases.

**Table 2:** Results of the four stages of the audit.

	“Pre-Protocol”		“Post-Protocol”		“Post-Proforma”		“+1 year”
	Admissions 1 Jun–31 Aug 2012	Audit 30 Jul–31 Aug 2012	Admissions 1 Sep 2012–2 Apr 2013	Audit 19 Oct–10 Nov 2012	Admissions 3 Apr 2013–1 Aug 2014	Audit 10 Jun–1 Aug 2013	Audit 28 May–20 Jul 2014
Patients admitted	392	136	1109	111	2848	120	119
Patients admitted >48 hours	201	73	481	61	1120	59	60
Median age in years (range)		53(20–87)		52 (17–95)		53 (16–90)	59 (28–95)
HIV test indicated		41		30		24	26
HIV test performed	18	9	98	11	262	20	17
Consent documented		0		5		15	10
**Overall testing rate**	**9.0%** ^a^ ^,^ ^b^	**12.3%**	**20.4%** ^a^	**18.0%**	**23.4%** ^b^	**33.9%**	**28.3%**
**Eligible testing rate**		**21.9%** ^c^ ^,^ ^d^		**36.6%** ^e^		**83.3%** ^c^ ^,^ ^e^	**65.4%** ^d^

Two-tailed Fisher’s exact test statistically significant at p<0.05 when comparing:

^a-b^ overall HIV testing rates between the “pre-proforma” and both “post-proforma” (a) and “post-protocol” (b) periods

^c-e^ proportion of HIV testing in eligible patients during case-notes audit between: “pre-proforma” and “post-protocol” (c); “pre-proforma” and “+1 year”; and “post-proforma” and “post-protocol” (e) periods

**Table 3:** Clinical presentations of patients eligible for HIV based on the Sheffield testing protocol.

Unexplained peripheral neuropathy	15
Leukoencephalopathy / atypical demyelination	13
Encephalitis	9
Transverse Myelitis	7
Space occupying lesion of unknown cause	7
Guillain-Barre syndrome	7
Myositis	5
Atypical dementia	4
Aseptic meningitis	3
***Other*:**	
Atypical optic neuritis ^a^	4
Unexplained ataxia ^b^	3
Complicated headaches ^c^	3
**Total**	**80**

^a^ as defined by the “atypical optic neuritis” protocol (see discussion section)

^b^ as a national ataxia centre, HIV testing was done on these patients after applying our departmental ataxia investigation panel

^c^ headaches with cranial nerve involvement or other neurological features

## Discussion/Conclusion

This is the first study investigating the effect of practical simple interventions to improve HIV testing uptake in neurology patients, whom are an important group as they include several conditions that can be result of acute seroconversion and chronic HIV infection. Compared to similar studies in other non-traditional settings (Table 1), we additionally measured the sustained effect of our interventions with a fourth stage of re-audit.

47.8% of our patients during the audit periods were eligible for HIV testing, compared to 15–28% of unselected acute medical admissions presenting with neurological problems [18, 33], which reflects the nature of acute neurological cases presenting to our neurology unit. The overall testing rate for all patients after introduction of the protocol, assuming a similar eligibility rate for testing throughout the post-protocol period, came to 19% which is comparable to other studies. Dissemination of the HIV testing protocol on its own increased uptake by a modest 15%, similar to previously reported studies [19, 22], although this is better than having no departmental UKNG-based protocol as a recent study of HIV testing in intensive care patients within our hospital Trust found a testing rate of <5% in eligible patients [34].

The proportion of eligible acute neurology and stroke patients who were tested for HIV doubled from the pre-protocol period to 20.4%, and continued to increase in the final audit period to 23.4%. The combined effect of our protocol, regular focussed teaching for admitting staff, and altering the admission clerking proforma increased testing uptake 3.7-fold to a maximum of 83%. The effect was sustained at a lower rate of 65% when audited one year later; this is comparable to 66.8% from the HINTS study [20]. This plateauing effect has been seen e.g. in the Croydon study, where after the initial intensive period of training clinical staff the rate peaked to 41.3% but then settled to 32.5% [18], partly attributed to the rapid turnover of staff in acute medical settings. (The testing rates during our case-notes audit period are almost twice as high as those reported in other acute settings probably due to the smaller numbers of cases [16–18, 27]). Since the “post-proforma” period, the HIV testing protocol is included as mandatory introductory material for junior doctors during our departmental induction session at the begin of their rotation. However, we found that one of the key interventional differences between the “post-proforma” and “+1 year” periods was that the focussed training of junior doctors regarding HIV testing was occurring towards the middle or end of their rotation in the “+1 year” period, rather than towards the beginning as was the case during the “post-proforma” period. The co-incident induction and focussed teaching during the post-proforma probably had a synergistic effect to increasing awareness of HIV testing amongst the new junior doctors. Therefore, to counter the plateauing effect, we will ensure that the focussed teaching returns to occurring within the first few weeks of the start of the rotation.

In the “post-protocol” period, the overall testing rate was 23.4% but in the audit period was slightly higher at 33.9%. This could be as a result of the more active interventions (focussed seminars and teaching sessions, awareness of the role out of the new proforma) in addition to the relatively passive intervention of disseminating the HIV testing protocol. These more dynamic interventions are more likely to have enthused the new house of junior medical staff during the audit period which is reflected in the high number of cases tested for HIV; such effects have been noted in other studies [17, 18]. This may also be the reason why 100% of patients approached consented to have their HIV status checked, as compared to 61.8%-82.8% acceptance rates in other studies [16, 20]. Taking the quoted estimated HIV prevalence of approximately 2 per 1000 in South Yorkshire, we would have expected to have 4 patients tested positive. No new HIV cases were encountered during our study period. This could be because:

1. acute general medical admissions are admitted at another city hospital, therefore acute patients with HIV clinical indicator diseases in whom the neurology component is not a dominant feature would by-pass our unit and be tested for HIV elsewhere;
2. patients admitted to district general hospitals with acute neurological problems are sometimes managed by neurologists without transfer to our unit;
3. despite the increase in HIV testing uptake through our interventions, over half of test-eligible patients were still not tested and therefore could have been missed.

Details of all patients who met local guidelines for HIV test but who were not tested were fed back to their respective neurology consultants for follow-up. It is important that screening continues, as one missed case is costly to public health funds [6]. Moreover, neurological admissions in general are varied and often result in many expensive tests, frequently with a poor return of positive diagnoses e.g. one component of a full vasculitis screen [35].

Barriers to testing for HIV perceived by frontline staff were not investigated in this study, but feedback revealed factors similar to previous studies: HIV stigma [36], variability of uptake amongst consultants [17], inadequate financial and workforce infrastructure [37], and perceived inadequate skill set [20]. The last factor was particularly apparent during the junior doctor teaching sessions, and it has been shown that despite HIV medicine being on the 2009 national core curriculum, over two-thirds of junior doctors still feel they need further training to confidently test for HIV [28, 38].

As with previous studies, our patients were mostly above the age of 50 years [18], in our case the upper limit of the age range being above 80 years as we did not dictate an age ceiling. Previous studies that did so eventually revised their age range of test eligibility: in the Leicester study, all patients newly diagnosed with HIV were above the age of 45 years [17], and three newly diagnosed HIV patients were above the age of 59 in the Croydon study [18]. This reinforces the importance of considering testing in older patients, as they are most likely to be late presenters and be missed for opportunistic HIV testing as they do not necessarily fall into “high-risk” categories [26, 33, 39].

The printed prompt for HIV testing (along with consent) in the neurology clerking proforma improved documentation of consent from 25% to 67.6%, but overall consent was poorly documented at an average of 35.1% whereas the UKNG mandate documentation of consent in every patient. A similar audit in another study had a rate of 58% [19].

1 in 8 of our test-eligible patients had conditions not specifically listed in the clinical indicator list. Three patients had unexplained ataxia; our hospital is a national centre of excellence for ataxia, and pure cerebellar dysfunction as a complication of HIV infection have been described [40]. Four patients were tested as they presented with atypical optic neuritis, as defined by local guidelines developed by our neuro-opthalmology service which recommends appropriate serology testing in such cases [41]. Three patients had complicated presentations of headache, including one case with meningism and low-grade fever, one case with cranial neuropathies and one with ophthalmoplegia not due to acute stroke. In other similar studies, 42–50% of patients who tested positive for HIV had a presentation not listed as an indicator condition, including presenting with acute headache [17, 18]. Given 10% of acute sero-conversion can present with meningitic or encephalitic features, there is rationale to include routine testing for HIV in acute non-primary headaches [29].

The data from overall HIV testing rates showed there were 23 HIV tests, approximately one a month, requested from stroke admissions. We did not audit HIV testing in acute stroke admissions in our study, but there are recommendations for HIV testing in all acute stroke patients, particularly in younger patients and cryptogenic cases [42]. We also did not audit HIV testing in neurology outpatients during our study as we were looking at acute admissions. Only one previous study looked at outpatient HIV testing in primary care and outpatient settings [20]. Our HIV testing protocol is readily available in our outpatients section, and our work has been presented within the department to consultants, registrars and junior doctors who work in clinics in order to reinforce HIV testing in the non-acute patients.

In conclusion, we have demonstrated that a combination of pragmatic interventions has led to a significantly sustained increase of HIV testing in eligible patients admitted to an acute neurology unit in an area with low HIV prevalence. We have shown that nearly one half of all acute neurology admissions merit HIV testing according to local guidelines. We strongly recommend all centres that admit acute neurology cases adopting similar interventions for opportunistic HIV testing in order to avoid missing neurological presentations of HIV. We also suggest extending interventions (along with audit) into neurology outpatient settings as we have to increase HIV testing in non-acute patients.
